# Optimizing Surface Hardness and Corrosion Resistance in 316L Stainless Steel via SMRT and Plasma Nitriding

**DOI:** 10.1021/acsomega.4c05906

**Published:** 2024-09-24

**Authors:** João R. de Barros Neto, Renan M. Monção, Muhammad Naeem, Brenda J. De Sousa Noleto, Maelson S. Nunes, Rafael M. Bandeira, Nicolau Apoena Castro, Michelle C. Feitor, Thércio H. De C. Costa, Maxwell S. Libório, Rômulo R. M. De Sousa

**Affiliations:** † Department of Materials Engineering, Federal University of Piauí, Teresina, Piauí 64049-550, Brazil; ‡ Postgraduate Materials Science and Engineering Program, Federal University of Piauí, Teresina, Piauí 64049-550, Brazil; ∥ Woman University of Azad Jammu & Kashmir, Bagh 12500, Pakistan; ○ Plasma Materials Processing Laboratory - LABPLASMA, Department of Mechanical Engineering, Federal University of Rio Grande do Norte, Natal, Rio Grande do Norte 59078-970, Brazil; ⊥ Postgraduate Program in Chemistry, Federal University of Piauí, Teresina, Piauí 64049-550, Brazil; ¶ School of science and technology, Federal University of Rio Grande do Norte, Natal, Rio Grande do Norte 59078-970, Brazil; # Department of Chemistry, Center for Natural Sciences, Federal University of Piauí, Teresina, Piauí 64049-550, Brazil; ∇ Postgraduate Materials Science and Engineering Program, Federal University of Rio Grande do Norte, Natal, Rio Grande do Norte 59078-970, Brazil; ◆ Postgraduate Program in Mechanical Engineering, Federal University of Rio Grande do Norte, Natal, Rio Grande do Norte 59078-970, Brazil; § Interdisciplinary Laboratory of Advanced Materials, Federal University of Piauí, Teresina, Piauí 64049-550, Brazil

## Abstract

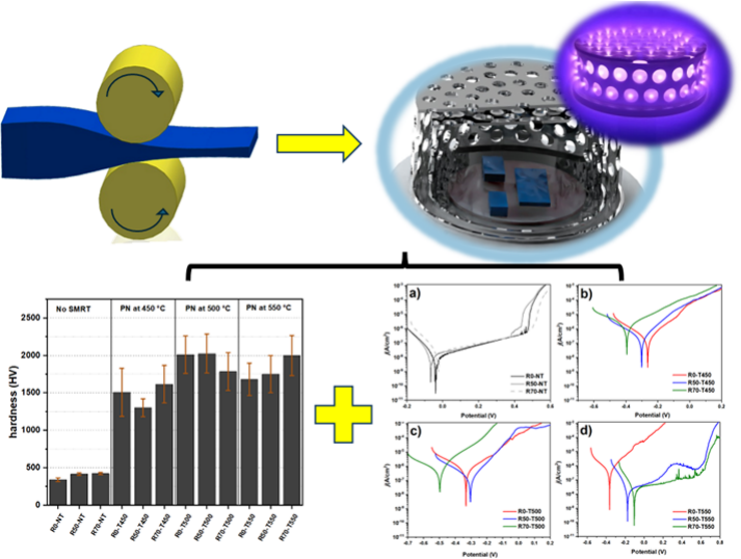

Increasing the mechanical strength of stainless steel is fundamental to expanding its applications and extending its useful life, especially due to its impressive corrosion resistance. However, many processes that make possible increasing the hardness and wear resistance of these steels also compromise their performance when they are subjected to corrosive environments. Because of the need to increase surface hardness without jeopardizing corrosion resistance, this study explores the impact of a surface mechanical rolling treatment (SMRT) with volumetric reductions of 50% and 70%, together with high-temperature plasma nitriding (PN) (450, 500, and 550 °C), on 316L steel. X-ray diffraction analysis analyzed the composition and structure of the resulting coatings. Mechanical strength was assessed using Vickers microhardness, while corrosion resistance was tested in a 3.5%-by-weight NaCl solution. The results indicate a substantial increase in surface hardness in all of the nitrided samples. Notably, the samples were subjected to rolling with 50% and 70% volumetric reduction, and subsequent nitriding at 550 °C showed a reduced impairment of corrosion resistance. This shows that the methodology adopted in this study, consisting of SMRT and PN, can increase the useful life and expand the applicability of stainless steels commonly used in various industry sectors.

## Introduction

Stainless steel (SS) alloys are widely used in the naval,^1,2^ biomedical,^3,4^ food,^5^ oil and gas mining industries,^6^ and other sectors where corrosion-resistant steels are needed.^7^ The 316L SS is an example of this due to the natural formation of a passive layer of Cr_2_O_3_ on the steel surface.^7^ This passive layer protects the surface of materials from possible degrading processes caused by corrosion and tribo-corrosion. On the other hand, stainless steels have low hardness and wear resistance when subjected to sliding contact due to their softness and susceptibility to adhesive and abrasive wear.^8−10^ These conditions limit the applicability of SS, requiring mechanisms capable of overcoming these problems.

Plasma surface modification technology is an alternative to overcome the structural limitations presented by SS. Conventional plasma nitriding (DCPN) and cathodic cage plasma nitriding (CCPN) are processes adopted to modify the surface of a metal without compromising the properties of its core.^9,11,12^ These processes promote the diffusion of nitrogen and precipitation of nitrides on the surface of the steel, forming a hard, wear-resistant layer.^13,14^ However, the surface hardness of the plasma-sprayed austenitic stainless-steel coatings is only about 300 HV, which makes them inadequate as wear-resistant coatings under severe friction conditions.^15^ This is because the nitriding processes in SS have to occur at low temperatures to not impair corrosion resistance^16,17^ due to the formation of a very stable and hard precipitate chromium nitride (CrN).^14^ This is the main reason for the existence and usability of these alloys.^18−21^

Scheuer et al. (2018) carried out plasma nitriding on AISI 420 steel, varying the treatment temperature from 200 to 600 °C for 4 h. The results showed the formation of chromium nitride precipitate phases in samples subjected to high-temperature treatments (*T* ≥ 450 °C). The films produced under these conditions showed high surface hardness, with values of around 1500 HV, while the samples nitrided at lower temperatures (200–300 °C) showed a surface hardness of around 250 HV. The authors point out that these surface conditions promote a significant increase in wear resistance but reduce corrosion resistance due to the decomposition of the solid solution αN^′^→ α – Fe + CrN.^22,23^

Another way to increase the surface hardness of stainless steels and consequently improve their tribological behavior is to use a surface mechanical rolling treatment (SMRT).^24−26^ This cold deformation technique consists of grain refinement, forming strain-induced martensite (ά) in the region closest to the surface.^27,28^ The effects of this transformation are an increase in mechanical strength and an undesirable reduction in ductility or toughness.^24^ Ralls et al. (2024) analyzed the tribological behavior of 316L steel subjected to the SMRT process. The authors reported the formation of ultrafine grains on the surface, increased hardness, and reduced steel wear when subjected to sliding contact.^26^ Huang et al. (2015) also reported the formation of induced martensite in the rolling process by rolling of the tool ball and the consequent increase in hardness.^25^ Tandon and Patil (2020) carried out rolling of 316l stainless steel with a reduction in thickness of 10% to 50% and subsequent electrochemical nitriding to evaluate mechanical strength and corrosion behavior. The results showed a gradual increase in the induced martensite phase with increasing deformation, a significant increase in surface hardness resulting from combining the two processes, and corrosion behavior similar to the untreated sample.^29^ In addition to these beneficial effects, Lei et al. (2021) and other authors reported a slight increase in corrosion resistance for SS subjected to SMRT.^24,30^

Therefore, this work proposes combining CCPN and SMRT at high temperatures to coat 316L SS with a coating capable of significantly increasing the surface hardness without causing severe damage to corrosion resistance. This will make 316L stainless steel more resistant to wear under mechanical stress and consequently expand the possibilities of applications for this steel in equipment that requires a corrosion-resistant steel with good mechanical strength.

## Methods

**Figure 1:**
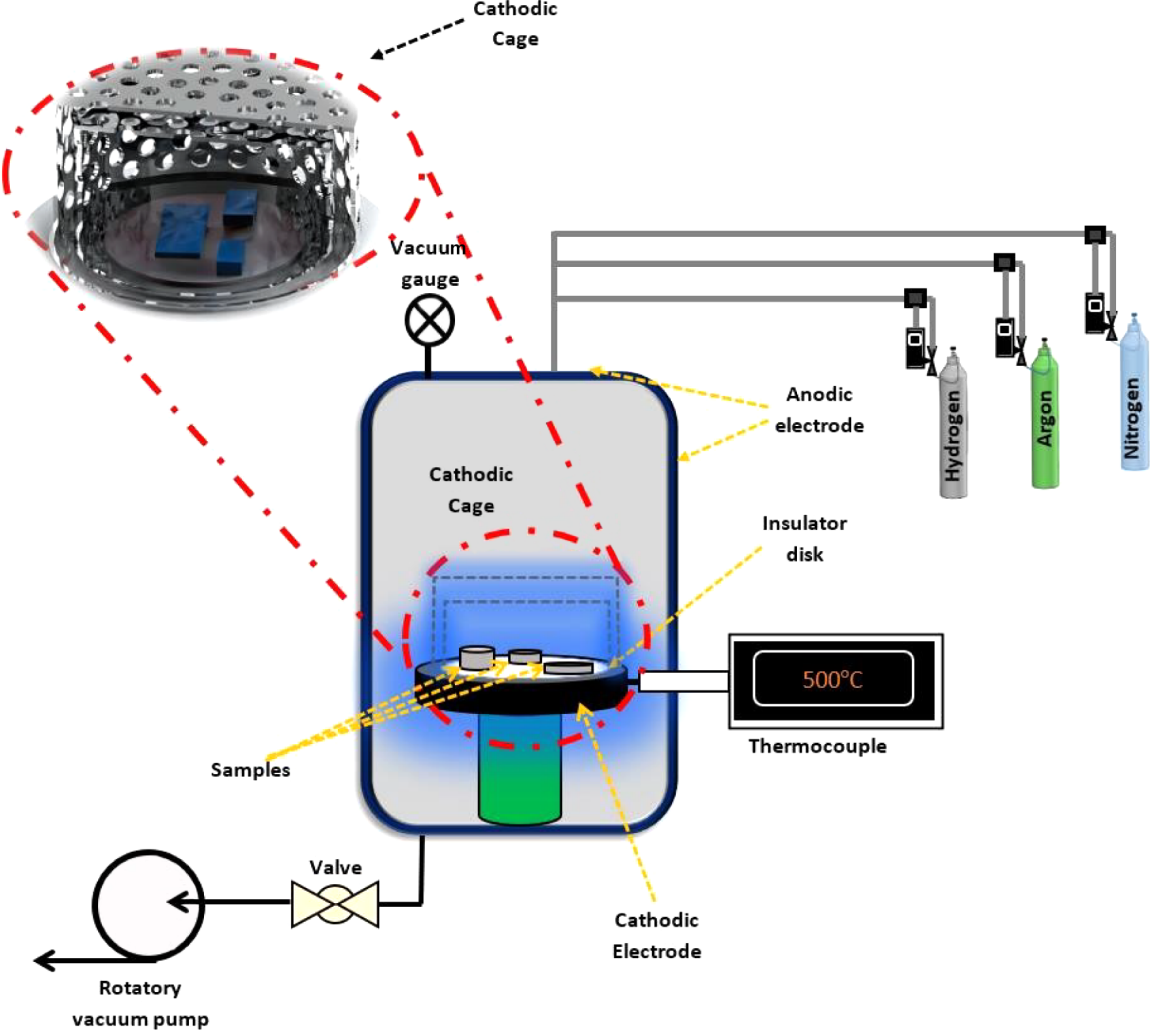
Schematic representation of the plasma deposition reactor with two cathode cages.

The surfaces of 316L austenitic steel, in at% (C = 0.03, Mn = 2.00, Si = 1.00, P = 0.04, S = 0.035, Cr = 16.5–18.5, Ni = 11–14, and balance Fe), with a diameter of 15 mm and a thickness of 6 mm were previously prepared with sanding and polishing, followed by an ultrasonic bath in acetone and ethanol for 10 min at room temperature, as reported in previous works.^31^ Some of these samples were cold-rolled with 50 and 70 mm reduction thicknesses to evaluate the influence of this stage on the final properties of the treated samples. The rolled and unrolled samples were subjected to cathodic cage plasma nitriding (CCPN) in an atmosphere of 75% H_2_ + 25% N_2_ using a rotary vane pump, which evacuated the chamber until 0.5 mbar before nitriding.^32^ Two cathodic cages of 316L steel were used in the nitriding processes. The largest cage has a 90 mm diameter and is 50 mm in height, while the smallest cage has a 70 mm diameter and is 40 mm in height. Figure 1 schematically illustrates the plasma reactor with the treated samples, following adaptation of ref (33). Both cages have 8 mm holes with a distance of 9.2 mm between the adjacent holes.

All samples were nitrided for 4 h, and only the source voltage was varied to increase the nitriding temperature, as detailed in Table 1.

**Table 1:** Nomenclature of the Samples Associated with the Rolling Conditions and the CCPD Temperature

Samples	Reduction by rolling (%)	Temperature CCPN (°C)	Source voltage (V)
R0-NT R0-T450	0	450	550
R0-T500		500	610
R0-T550		550	680
R50-NT R50-T450	50	450	550
R50-T500		500	610
R50-T550		550	680
R70-NT R70-T450	70	450	550
R70-T500		500	610
R70-T550		550	680

For XRD analysis, a Bruker D2 Phase diffractometer with copper Kα radiation (λ = 1.54 Å), 2θ intervals between 40° and 100°, 0.01° step, 10 mA current, and 30 kV voltage was used. The hardness of the films subjected only to the SMRT process was measured using a Rockwell indentation hardness tester (HRC) with a load application of 150 kgf. The microhardness tests were performed according to ASTM E92 standards using a Vickers pyramidal tip in an INSIDE microdurometer model ISH – TDV 1000 A-B.^34^ Five hardness measurements were taken on each sample to obtain the average. Cross-section microscopy was carried out with a Bel Photonics MTM-1 microscope. Corrosion tests were performed with a three-electrode system (3.5 wt % NaCl solution) with a platinum wire as the counter electrode in Autolab potentiostat/galvanostat equipment for 2 h. Potentiodynamic polarization measurements started at −650 mV and were interrupted when the current reached 1 mA/cm^2^.

## Results and Discussion

**Figure 2:**
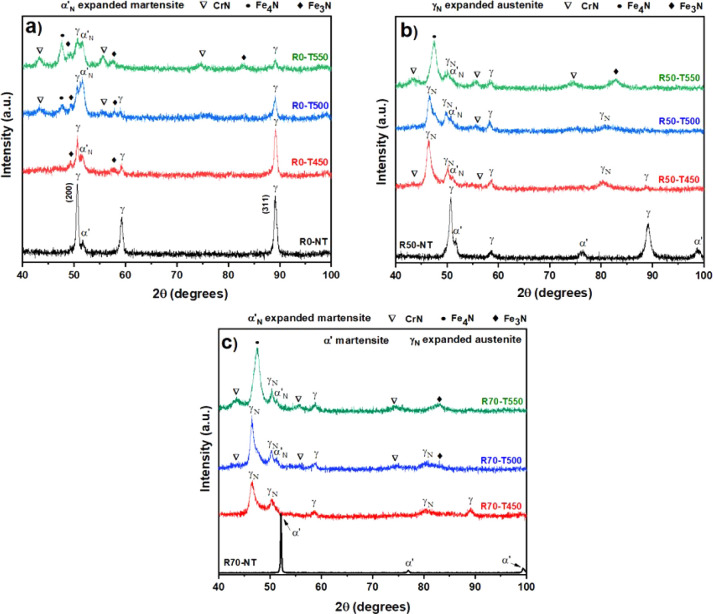
Diffractograms of the samples submitted to the SMRT and plasma nitriding processes without SMRT (a), SMRT (50%) (b), and SMRT (70%) (c).

Figure 2 shows the X-ray diffraction results of the rolled and nitride samples. The untreated 316L sample (R0-NT) results show the predominant presence of Fe-γ (austenite), characteristic of the steel purchased. This structure may result from an annealing heat treatment process, which is necessary to improve the corrosion resistance of stainless steel.^35^ A small Fe-α’ (deformation martensite), which can be attributed to surface hardening caused by the grinding and mechanical polishing process, is also observed.^36,37^

The unrolled samples and those only nitrided at 450, 500, and 550 °C showed a progressive reduction of the Fe-γ phase and the growth of other phases due to the interaction of the sample surface with the nitriding atmosphere.

Notably, nitriding at 450 °C resulted in expanded martensite α’N and Fe3N appearance.^32^ The increase in nitriding temperature to 500 °C further enhanced the α’_N_ phase peak and the appearance of the Cr_2_N and Fe_4_N precipitates commonly in high-temperature nitriding processes.^38,39^ The R0-T550 sample, due to the high nitriding temperature, showed greater intensity of the chromium nitride and iron nitride peaks, highlighting the significant impact of the nitriding temperature on the formation of different phases.

The presence of nitride precipitates significantly enhances the mechanical strength of stainless steels, with the formation of Fe_4_N and CrN being the main contributors. However, it is important to note that this enhancement comes at the cost of reducing the material’s corrosion resistance. The formation of chromium nitrides results from the reduction of chromium in the solid solution of the material structure.^40,41^ According to Patel et al. (2022), the increase in plasma nitriding temperature above 400 °C decreases the fraction of the Fe_2_N phase. The decomposition of the Fe_2_N phase and subsequent diffusion of nitrogen atoms into the substrate produced other nitrides such as Fe_3_N, Fe_4_N, and CrN.^42^ This may justify the absence of Fe_2_N in the composition of the layers produced with the high temperatures adopted.^43^

Figure 2b,c shows the diffractogram of the rolled samples with 50% and 70% reduction, respectively. The samples submitted to SMRT showed the formation of the γ_N_ phase, which reduced with the increase in temperature. The increase in the incorporation of nitrogen into the solid solutions increased the nitride fraction, forming a mixed coating composed of a solid solution with nitrogen and chromium and iron nitrides, as can be observed in the samples rolled with 50% and 70% reduction and temperatures of nitriding at 500 °C and 550 °C. (44)

The samples without plasma treatment (R50-NT and R70-NT) show a reduction of the austenite phase and the appearance of formation martensite. Figure 2c shows that the SMRT process with 70% reduction (dark curve) caused strong plastic deformation capable of forming the deformation martensite phase to a great depth. This resulted in the nondetection of the austenitic phase shown in the other non-nitrided samples. However, the plasma nitriding process led to the appearance of the expanded austenite phase and the formation of iron nitride and chromium nitride as the treatment temperature increased. The formation of expanded austenite occurs due to stress relaxation during the thermochemical nitriding process. R50-T550 shows that the expanded austenite has saturated to the point of precipitation in CrN. The diffractograms of the samples rolled with a 70% reduction and plasma nitrided are shown in Figure 2c. The phases found in these samples are similar to those observed in the samples rolled with a 50% reduction. However, the peaks resulting from the nitriding process are more intense in the 70% rolled samples.

**Figure 3:**
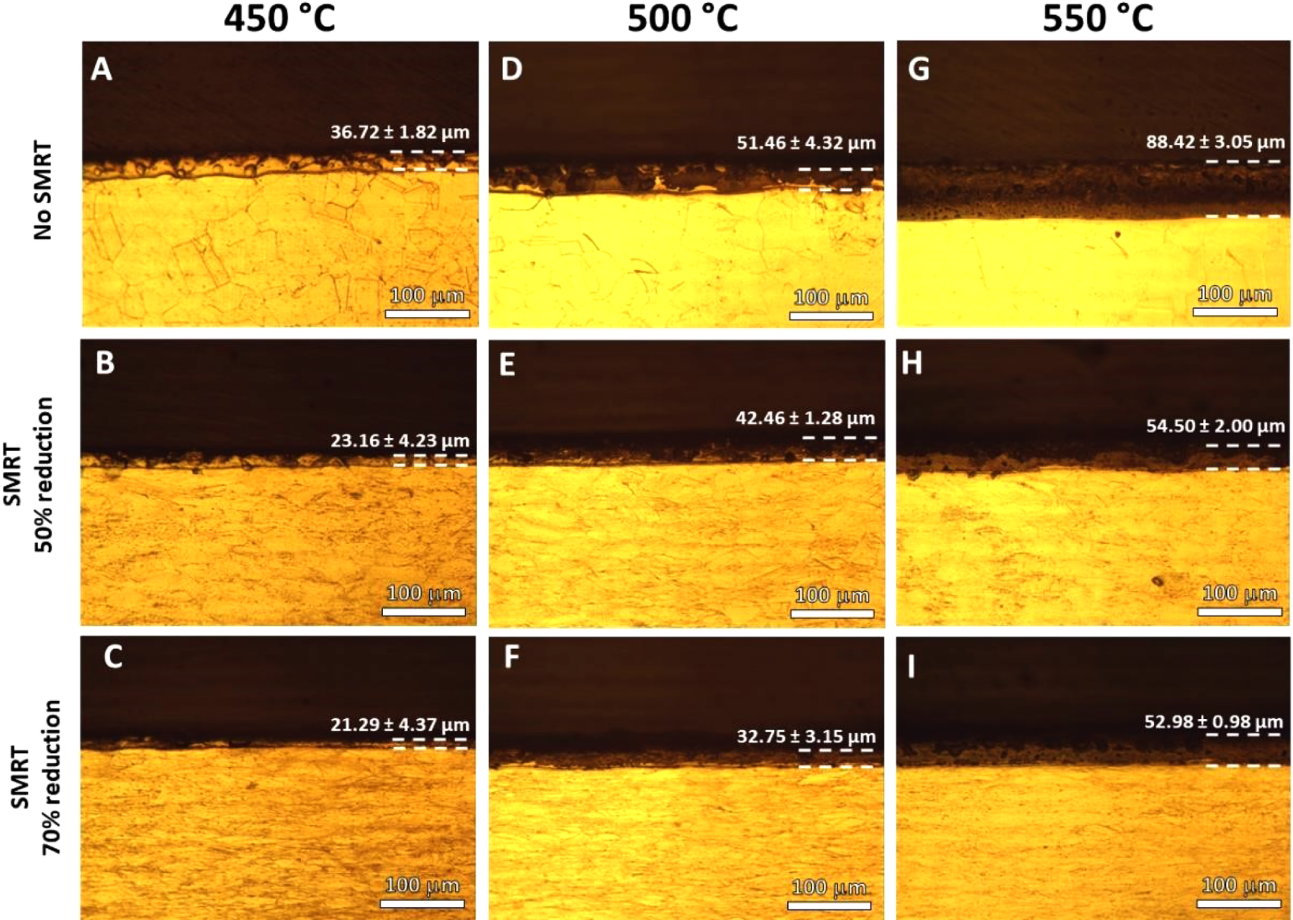
Cross-section optical microscopy of the analyzed samples: (A) R0-T450, (B) R50-T450, (C) R70-T450, (D) R0-T500, (E) R50-T500, (F) R70-T500, (G) R0-T550, (H) R50-T550, and (I) R70-T550.

The cross-sectional micrographs shown in Figure 3 allow for visualization of the surface layers. It can be seen that there was an increase in the thickness of the layers as the nitriding temperature increased in all substrate conditions (with and without SMRT). The more apparent contrast of the coatings produced at 450 °C is characteristic of the solid solution phases already revealed in the diffractograms in Figure 2. However, it has been reported in several studies that at this temperature, the formation of chromium nitride precipitates has already occurred, which are identified by the darker regions.^45−47^ The chromium nitride phases were not detected by the X-ray diffraction carried out on the samples nitrided at 450 °C because the Bragg–Brentano configuration, unlike the shallow angle incidence configuration, obtains information more profound in the layer that presents reflections that can suppress those occurring in the more superficial crystalline structure. Therefore, darker phases in the outer regions may justify the nondetection of chromium nitrides by XRD analysis, as seen in Figure 2.

The SMRT process reduced the thickness of the nitrided layer. The processes caused surface structural changes that resulted in changes in the formation kinetics of the nitrided layers. In addition to contributing to the formation of different crystalline phases, as observed in the previous diffractograms, the SMRT process reduced the thickness of these layers resulting from the plasma nitriding process.

**Figure 4:**
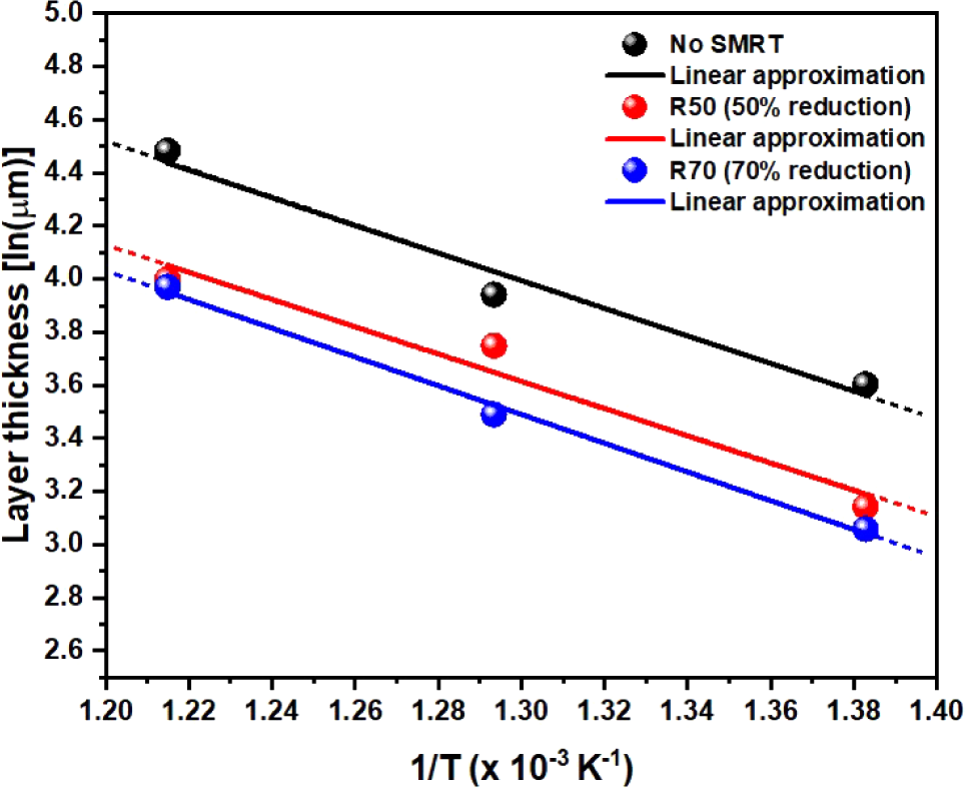
Arrhenius plot formed by a linear approximation relating the thickness of the layers obtained to the treatment temperature.

Figure 4 shows the Arrhenius plot of the thickness of the nitrided layer. High temperatures and low activation energy favor higher velocity constants and thus accelerate the chemical reaction.^48^ Therefore, it can be seen that for higher temperature values, i.e., low 1/T, surface modification is more straightforward (greater thickness). The displacement of the curves defined by the points assigned to conditions R50 and R70 shows that the SMRT process causes the structure on the surface of rolled 316L steel to require more energy for the appropriate chemical reactions.

**Figure 5:**
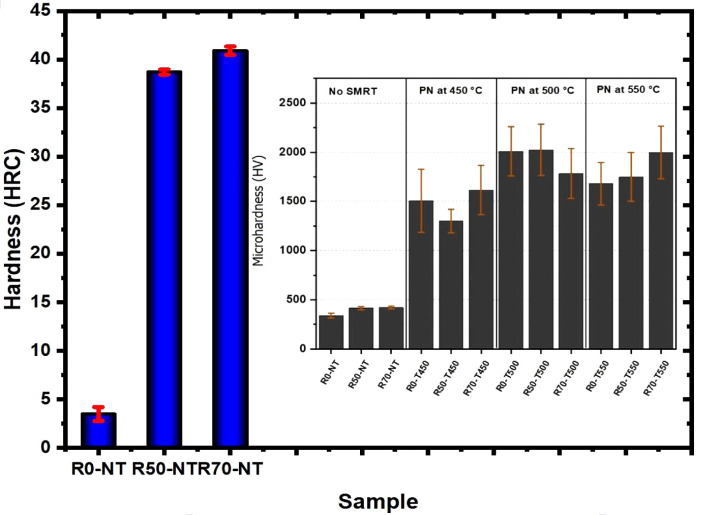
Rockwell hardness and Vickers microhardness results of the samples subjected to SMRT and plasma nitriding.

Figure 5 shows the hardness results of the untreated rolled samples in Rockwell C and the surface microhardnesses of the rolled, treated, and untreated samples on the Vickers scale. As reported in the literature,^27,28^ the hardness of metallic materials tends to increase after a cold working process due to factors such as work hardening and changes in grain size. In this case, the hardness in the macroscale increased, ranging from 3.5 HRC to 39 and 41 HRC, when the rolling percentage varied from 0%, 50%, and 70%, respectively. However, it is worth mentioning that when measuring the hardness in microscale (Vickers), the variation in the hardness of the untreated samples is minimal; this is due to the size of the deformed area during the microhardness test since the indentation must have been measured within the grain boundary, which makes it difficult to identify the change in hardness due to cold deformation. However, measuring microhardness is essential to identifying the effects of nitriding on samples, as it is a surface treatment. The untreated sample showed a surface hardness of 340 ± 25 HV, and the rolling processes with 50% and 70% reduction showed a slight increase in surface hardness compared to the R0-NT sample. However, this increase does not show significant changes due to the measurement errors attributed to the respective standard deviations. The nitrided samples showed a significant increase in hardness for all treatment conditions, reaching average values of 2000 HV in samples nitrided at 500 and 550 °C. Samples nitrided at 450 °C showed slightly lower hardness values than those obtained at 500 and 550 °C. The high standard deviation of the hardness measurements taken on the nitrided samples shows that the temperature variation between 450 and 550 °C did not offer any significant advantages regarding the material’s mechanical strength. The high dispersion of the measurements may be due to the heterogeneity of the films in terms of the distribution of chromium and iron precipitates throughout the volume of the nitrided layer.

**Figure 6:**
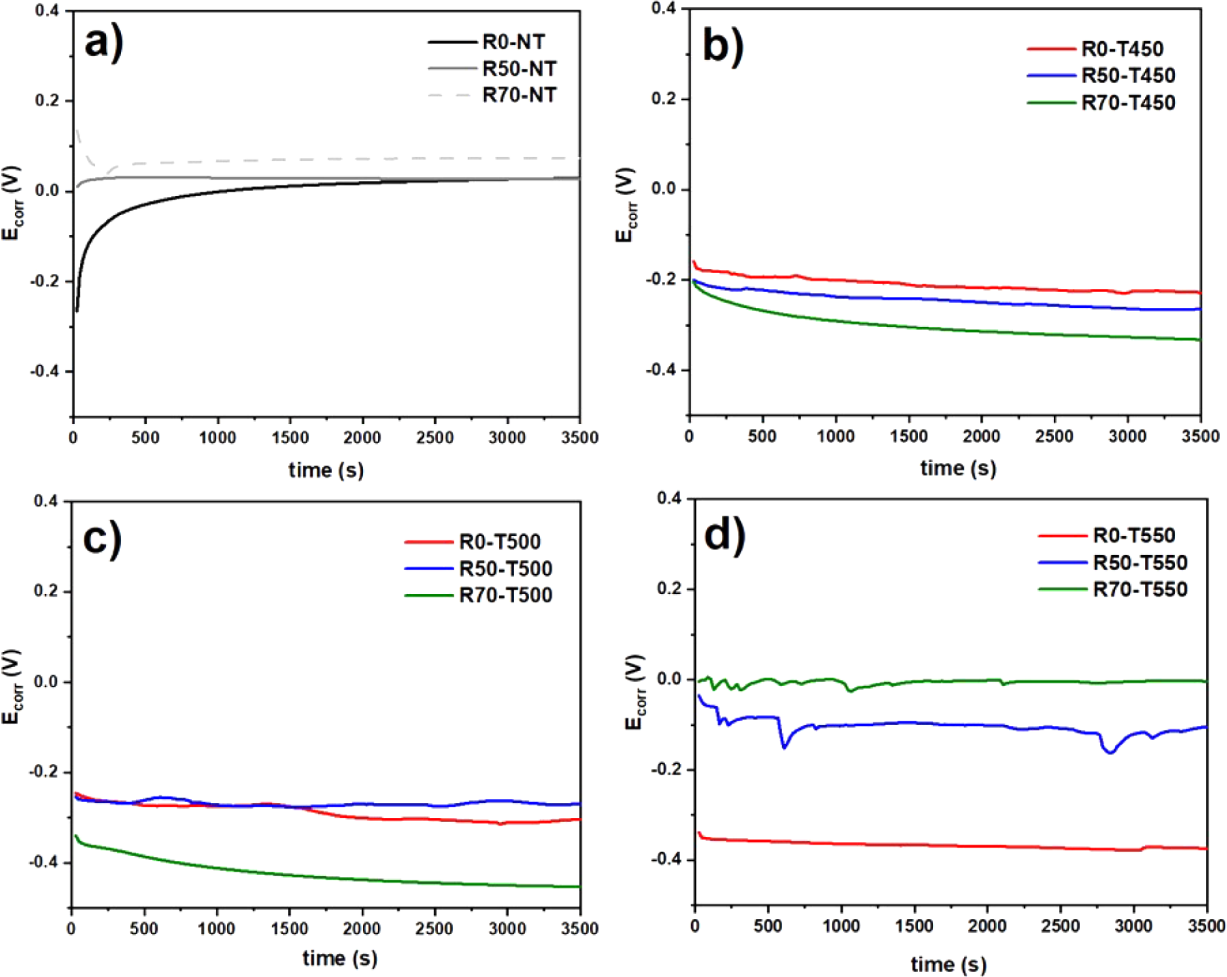
Evolution of the OCP of samples subjected to SMRT and plasma nitriding: (a) untreated samples and nitrided samples at (b) 450, (c) 500, and (d) 550 °C.

The response of the 316L stainless steel samples with and without treatments is analyzed through variation in the open circuit potential (OCP) as a function of the time the electrodes are exposed to the electrochemical solution. Figure 6a shows that the OCP vs time curves of the untreated sample showed the most negative potential values over the first few minutes of monitoring, indicating greater electrochemical activity when compared to the samples subjected to the SMRT process.^49^ There was an upward trend in the open circuit potential at the start of the monitoring period for the R0-NT sample and a much lower one for R50-NT, indicating that the passive film will probably become thicker over time and samples subjected to SMRT showed greater stability during the test up to 2000 s, which is concluded by the higher *E*_corr_ values in this period.^50,51^

However, the R70-NT sample showed better behavior throughout the test (up to 3500 s), reaching a potential shift to a more positive value of 0.07 V compared to the unrolled sample with 0.03 V. Although steel with martensitic characteristics naturally has a lower corrosion resistance, the opposite effect is due to several factors. The R50-NT and R70-NT samples, which had more deformation martensite, became more susceptible to corrosion before the start of the OCP corrosion test and potentiodynamic polarization curves. As a result, the oxide formed on the surface of these samples induces a more positive potential than the sample with austenite characteristics.^52^

According to Liu et al. (2010), the gradual decrease in corrosion potentials observed in the initial test period with the R70-NT sample may result from the dissolution of the thin corrosion layers on the electrode surface when exposed to atmospheric air. This means the electrode must remain in contact with the solution longer before starting the corrosion test.^53^

Figures 6b–d shows that the nitriding process at high temperatures reduces the corrosion resistance of 316L stainless steel. Samples nitrided at 450 °C showed worse behavior with increasing volume reduction by rolling. Samples nitrided at 500 °C showed even worse responses, reaching a corrosion potential of −0.33 V for R70-T450. On the other hand, samples nitrided at 550 °C showed improvements with increased rolling. The presence of peaks in the OCP curves of the R50-T500 and R70-T550 samples indicates that surface activity with the solution occurred, characterizing localized corrosion such as pitting. However, the subsequent formation of a passivating layer stabilized the corrosive behavior.^52−54^

**Figure 7:**
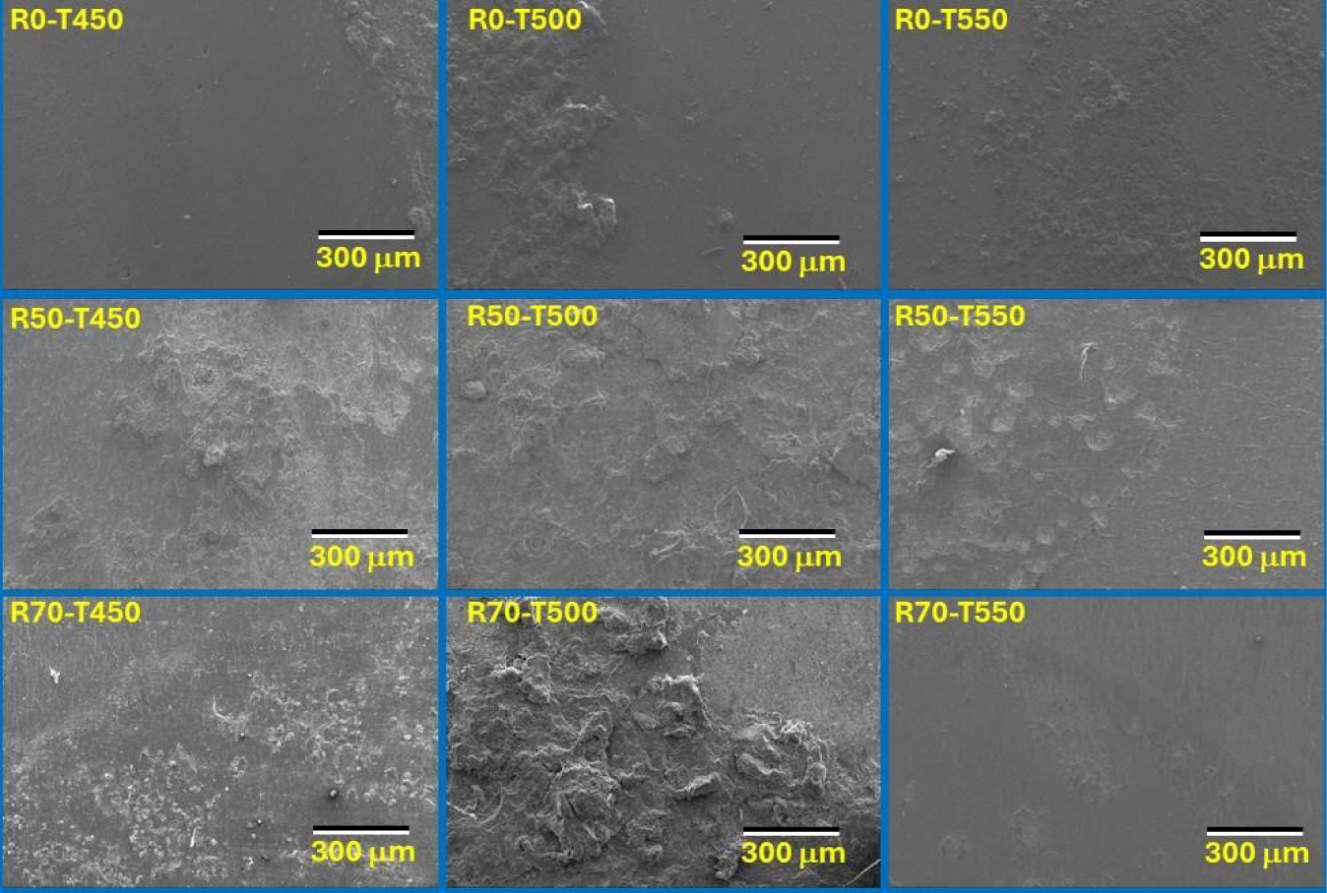
Morphology of treated samples after the corrosion resistance test.

Figure 7 shows the results and surface morphologies of the nitrided samples. It can be seen that the rolled samples treated at 450 and 500 °C present a corrosion process with surface delamination, while for the samples treated at 550 °C, a possible increase in corrosion resistance is observed, with pitting corrosion being selected for the samples, as described above. The increase in corrosion resistance is due to the formation of a layer rich in Fe_4_N and a reduction in phases such as expanded martensite, which provides the formation of a passivating layer, stabilizing the corrosive behavior.^44,46,52−55^

**Figure 8:**
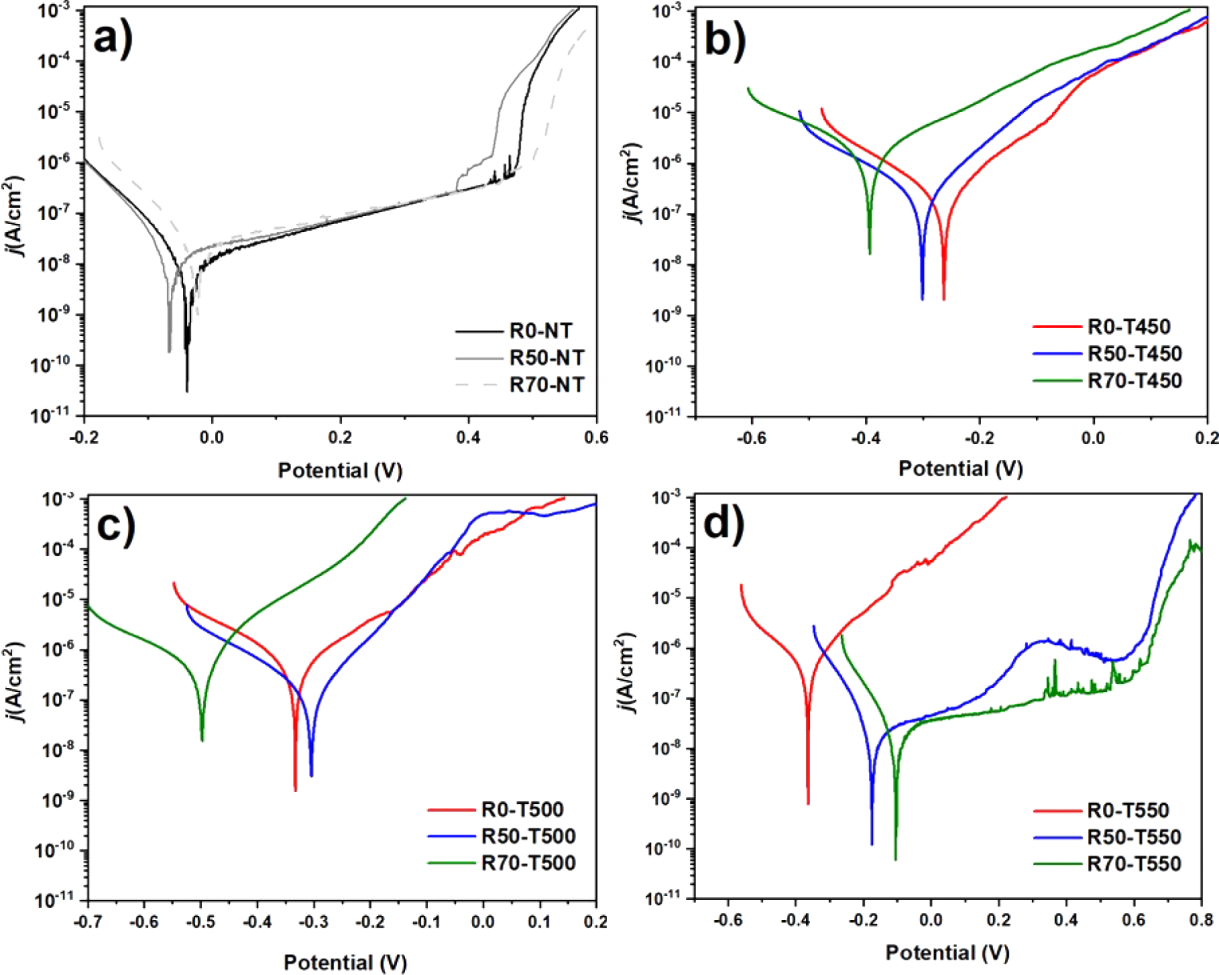
Potentiodynamic polarization curves of samples subjected to SMRT and plasma nitriding: (a) untreated samples and nitrided samples at (b) 450, (c) 500, and (d) 550 °C.

Figure 8 shows the potentiodynamic polarization curves resulting from the test carried out in a 3.5 wt % NaCl solution, which analyzes the corrosive behavior in relation to the current density produced during the test. Figure 6a shows the SMRT process’s influence on the samples’ corrosive behavior.

The shift to the right of the curve obtained with sample R50-NT indicates that the SMRT process with a 50% reduction makes the sample surface more susceptible to degradation in a corrosive environment. This agrees with the result observed in the diffractogram, in which rolling reduces austenite on the sample surface. The R70-NT sample, on the other hand, does not show a significant change from that of the R0-NT sample.

The sudden increase in current density in the high-potential region is due to the effect of pitting corrosion. Due to chloride ions’ action, localized corrosion starts at 0.46 V and is observed in sample R0-NT.^16,55^ This localized corrosion shifted to a more positive potential in sample R70-NT, showing that it remains more stable over a wider range of potential variations than the R0-NT and R50-NT samples.^50^

All of the nitrided samples showed a lower E_corr_ corrosion potential than the untreated samples with and without rolling. This means that nitriding at high temperatures makes 316L steel more susceptible to the onset of passive layer formation. In addition, the samples nitrided at 450 and 500 °C showed higher current density growth rates than those without plasma nitriding. This can be explained by the formation of residual stresses in the grain boundaries caused by nitriding, making the corrosion resistance more fragile.^53^ Despite the reduction in corrosion resistance of the nitrided samples, it can be seen that in the set of samples nitrided at 500 °C, the SMRT process with 70% reduction showed an increase in corrosion resistance. Similarly, Figure 6d shows that the SMRT process made surfaces less susceptible to corrosion than the sample nitrided at 550 °C without rolling (R0-T550). Despite showing some metastable pitting points during the corrosion test, the R70-T550 sample (−0.1 V) showed a corrosion potential closer to the R0-NT sample (−0.05 V) and lower current density values from 0.16 V onward.^56^

Table 2 shows the characteristic values of the corrosive behavior of the samples extracted from the potentiodynamic polarization curve. The polarization curves show that the steel with martensitic characteristics has a better corrosion potential, although it is not significantly higher than the sample with austenite characteristics. From the point of view of corrosion resistance, the R0-NT sample is less susceptible to corrosion due to its lower corrosion current density. The corrosion current density of the R0-NT sample is three times lower than that of the R70-NT sample (Table 2). Furthermore, the difference in corrosion current density between the R0-NT and R70-NT samples is more significant than the difference in corrosion potential between them.

**Table 2:** Results of the Corrosion Resistance Test in AISI 316 Subject to SMRT and Plasma Nitriding in 3.5 wt % NaCl Solution

Samples	*E*_corr_ (V)	*j* (mA cm^–2^)	*E*_pit_ (V)
R0-NT	–0.039	0.012	0.460
R50-NT	–0.067	0.010	0.380
R70-NT	–0.023	0.037	0.505
R0-T450	–0.263	0.271	-
R50-T450	–0.300	0.289	-
R70-T450	–0.394	3.180	-
R0-T500	–0.333	0.768	-
R50-T500	–0.303	0.166	-
R70-T500	–0.497	0.530	-
R0-T550	–0.365	0.641	-
R50-T550	–0.175	0.011	0.567
R70-T550	–0.104	0.036	0.603

The measurements showed that the SMRT process with a 70% reduction improved corrosion resistance compared to the other nitrided samples, with values of *E*_corr_ = −0.104 V and *j*_corr_ = 0.036 A cm^–2^. The samples nitrided at 450 and 500 °C showed an increase in current density as a function of the percentage of volumetric reduction by the SMRT process. However, these samples did not reveal pitting corrosion, unlike what can be observed in the non-nitrided samples, the R50-T550 samples, and the R50-T550 and 70-T550 samples.

In addition, the low surface roughness of the samples must be the factor responsible for the more positive pitting potential observed for the R70-NT sample. Since the surface treatment minimizes the structural defects, the pitting potential may change between the samples, although increasing it too much is impossible. There was no significant increase in the pitting potential, especially considering the sample preparation conditions.^57^

## Conclusions

The study of the influence of the rolling process previously applied to 316L steel nitrided at high temperatures (450, 500, and 550 °C) led to the following conclusions as consequences of the results presented:

- The rolling process produces surface structural modifications in the steel matrix, resulting in defects that influence the diffusion and formation of nitride precipitates in the subsequent nitriding process, as described by the X-ray diffraction results.
- The reduction in thickness of the nitrided layers with the percentage increase in volume reduction by rolling influenced the mechanical strength measured by Vickers microhardness.
- The corrosion results show that the rolling process was damaging for the samples nitrided at 450 and 500 °C, but the samples nitrided at 550 °C and rolled with 50 and 70% volumetric reduction showed better stability in a corrosive environment compared to the other nitrided samples.
- The SMRT process applied before high-temperature plasma nitriding proves viable when aiming to increase the surface mechanical strength of 316L stainless steel without seriously damaging its corrosion resistance.
